# Legumain/pH dual-responsive lytic peptide–paclitaxel conjugate for synergistic cancer therapy

**DOI:** 10.1080/10717544.2022.2081380

**Published:** 2022-05-31

**Authors:** Shanshan Zheng, Yue Cai, Yulu Hong, Yubei Gong, Licheng Gao, Qingyong Li, Le Li, Xuanrong Sun

**Affiliations:** Collaborative Innovation Center of Yangtze River Delta Region Green Pharmaceuticals & College of Pharmaceutical Science, Zhejiang University of Technology, Hangzhou, China

**Keywords:** Peptide–drug conjugate, legumain-responsive, pH-responsive, synergistic cancer therapy

## Abstract

After molecule targeted drug, monoclonal antibody and antibody–drug conjugates (ADCs), peptide–drug conjugates (PDCs) have become the next generation targeted anti-tumor drugs due to its properties of low molecule weight, efficient cell penetration, low immunogenicity, good pharmacokinetic and large-scale synthesis by solid phase synthesis. Herein, we present a lytic peptide PTP7-drug paclitaxel conjugate assembling nanoparticles (named PPP) that can sequentially respond to dual stimuli in the tumor microenvironment, which was designed for passive tumor-targeted delivery and on-demand release of a tumor lytic peptide (PTP-7) as well as a chemotherapeutic agent of paclitaxel (PTX). To achieve this, tumor lytic peptide PTP-7 was connected with polyethylene glycol by a peptide substrate of legumain to serve as hydrophobic segments of nanoparticles to protect the peptide from enzymatic degradation. After that, PTX was connected to the amino group of the polypeptide side chain through an acid-responsive chemical bond (2-propionic-3-methylmaleic anhydride, CDM). Therefore, the nanoparticle (PPP) collapsed when it encountered the weakly acidic tumor microenvironment where PTX molecules fell off, and further triggered the cleavage of the peptide substrate by legumain that is highly expressed in tumor stroma and tumor cell surface. Moreover, PPP presents improved stability, improved drug solubility, prolonged blood circulation and significant inhibition ability on tumor growth, which gives a reasonable strategy to accurately deliver small molecule drugs and active peptides simultaneously to tumor sites.

## 1. Introduction

Paclitaxel (PTX), as one of the most widely used chemotherapeutic agents, has been extensively used for the treatment of lung, breast and ovarian cancers (Kubota et al., 2014; Fleming et al., 2017; Kim et al., 2017). However, the limitations of PTX, such as highly hydrophobic, drug resistance as well as severe side effects have hindered its clinical applications (Singla et al., 2002). In order to enhance the physical and chemical properties of small molecule drugs, nano drug delivery systems (NDDSs) have been eagerly developed that feature passive or active targeting, enhanced stability in blood, improved bioavailability, high tumor accumulation and reduced toxic side effects (Davis et al., 2008; Song et al., 2019; Mirza & Karim, 2021). Although lots of researches about assembling chemotherapy drugs into nanoparticles have been explored (Yang et al., 2012; Wu et al., 2014; Piao et al., 2019), therapeutic peptides were seldom used to conjugate with chemotherapy drugs. Peptides are considered as a series of amino acids and penetrate easily into the tissue (Bolhassani et al., 2017). PTP-7 (FLGALFKALL) is a cationic lytic peptide which possesses primary target site on the cell membrane. Anticancer activities have been demonstrated in three thermodynamic steps: (1) the electrostatic attraction of cationic peptides to anionic cell membranes; (2) the accumulation of peptides on the cell surface; (3) the insertion of aggregated peptides into cell membranes to cause cell lysis (Tu et al., 2009; Chen et al., 2012a; Rafferty et al., 2016). Similarly, it can cause severe tissue damages due to its inability to make a distinction between tumor cells from normal cells (Chen et al., 2012b). Compared to small-molecules, PTP-7 is prone to degradation by proteases and has short half-life in the circulation so that have not been successfully applied to clinical cancer treatments (Tu et al., 2007; Luo et al., 2020).

Peptide–drug conjugates (PDCs) are special drug delivery system which comprises a therapeutic peptide, a small molecule drug and a linker. Compared with single drug loaded drug delivery system, PDCs can enhance anti-tumor effect, reduce drug resistance and modified with virous linkers (Deng et al., 2021; Zhu et al., 2021). Therefore, constructing PTX/PTP-7 co-loaded polymeric nanoparticles could be a creative strategy which can overcome the above shortcomings. Given weak acidic pH is a special feature of tumor microenvironment, acid-sensitive linkages play an important role in establishing prodrugs with efficient transformation in tumor (Wang et al., 2011; Saadat et al., 2021). Li et al. utilized CDM as an acid-sensitive linkage to control prodrug release in tumor (Li et al., 2016). Moreover, Ding et al. designed a PEGylated PTX prodrug using acid-sensitive acetone-based acyclic ketal as the linkage which showed good antitumor efficacy (Mu et al., 2020). Legumain, a lysosomal/vascular cysteine protease, is strictly specific to the hydrolysis of peptide bonds with asparagine or aspartic acid (Morita et al., 2007). Legumain was demonstrated overexpressing in various cancers, such as breast cancer, colorectal cancer, and prostate cancer, whereas rarely expressed in normal tissues (Ohno et al., 2010; Haugen et al., 2015). According to the unique function and characterization of legumain, several legumain-targeted NDDS have been explored to increase drug bioavailability and minimize or eliminate side effects (Liu et al., 2003; Lin et al., 2015; He et al., 2018).

**Figure 1.**
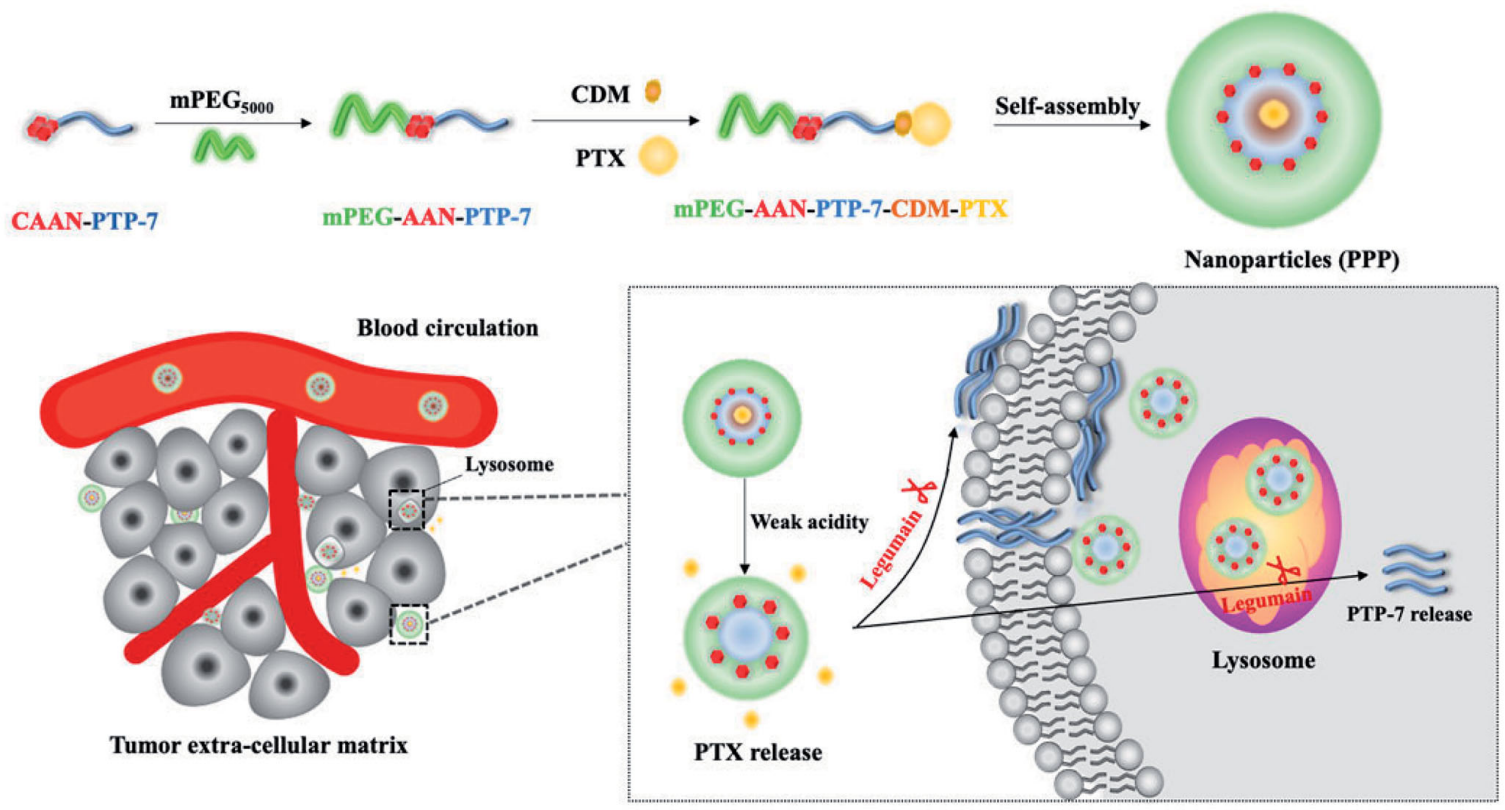
Schematic illustration of PTX and therapeutic peptide PTP-7 co-delivery nano-system could both response to tumor acidity and highly expressed legumain environment.

In view of their own disadvantages of both therapeutic peptide PTP-7 and small molecule drug PTX, overcoming one individual obstacle was not sufficient to optimize therapeutic outcomes. Thus, we designed weak acidic-responsive and legumain-cleavable PTX/PTP-7 co-loaded nanoparticles (mPEG-PTP-7-PTX, PPP). To be specific, PTP-7, as hydrophobic segments, was conjugated with maleimide-polyethylene glycol by using four amino acid CAAN, a substrate of legumain (Liu et al., 2003), and CDM-PTX was connected to the side chain amino group of PTP-7 via ring-opening and addition reaction. Once PPP encounters the acidic tumor microenvironment, PTX will directly fell off PPP and released in the tumor matrix or released in the cells after PPP endocytoses into lysosomes. Then, the legumain-sensitive site of PTX-released PPP (mPEG-PTP-7) was exposed and cut by legumain overexpressed in breast carcinoma and colorectal carcinoma, such as MCF-7, 4T1, and HCT116 cancer (Liu et al., 2014; Zhang & Lin, 2021), and finally releases PTP-7 to kill the tumor cell by cracking cell membrane (Figure 1). Our expectations are possible that PPP enhances the water solubility of PTX and enables PTX to target precise delivery to the tumor cells. What is more, PPP could protect PTP-7 from being degraded and prolong its half-life in the blood, subsequently achieve efficient synergistic anti-tumor effect of PTX and PTP-7.

## 2. Materials and methods

### 2.1. Materials

Peptides AAN-PTP-7 (AANFLGALFKALL) with purity ≥97% were purchased from ChinaPeptides Co., Ltd. (Shanghai, China). Methoxy poly(ethylene glycol) (Mn = 5000 g/mol, mPEG_5000_-OH) was purchased from Aladdin Bio-Chem Technology Co., Ltd. (Shanghai, China). Tris (2-carboxyethyl) phosphine hydrochloride (TCEP), N-maleoyl-β-alanine 1-ethyl-3-(3-dimethylaminopropyl) carbodiimide hydrochloride (EDC·HCl), 4-(dimethylamino)-pyridine (DMAP), and PTX were all obtained from Energy Chemical (Shanghai, China). Tetrahydrofuran (THF) was purchased from Titan (Shanghai, China). Dulbecco’s modified Eagle medium (DMEM) (high glucose) cell culture medium, RPMI 1640 medium, penicillin/streptomycin stock solutions (PS), fetal bovine serum (FBS), and trypsin were all purchased from Gibco BRL (Gaithersburg, MD). 1,1′-Dioctadecyl-3,3,3′,3′-tetramethylindocarbocyanine perchlorate (Dil) was purchased from Beyotime Biotechnology (Shanghai, China). Hoechst 33342 and 4% paraformaldehyde were supplied by Solarbio Science & Technology Co., Ltd. (Beijing, China). Taxol injection was available from Yangtze River Pharmaceutical (Group) Co., Ltd. (Taizhou, China).

### 2.2. Methods

#### 2.2.1. Synthesis of PPP nanoparticles

Maleimide acid was attached to mPEG by esterification reaction. mPEG-OH (1 mmol), Mal-COOH (4 mmol), EDC·HCl (4 mmol), and DMAP (0.1 mmol) were dissolved into dry dichloromethane (DCM). The solution was stirred at room temperature for 72 h under nitrogen atmosphere. Then, the mixture was diluted with DCM, washed with HCl (0.01 M), saturated sodium bicarbonate and saturated brine in turn, then dried over anhydrous sodium sulfate and evaporated, precipitated in excessive ice diethyl ether and dried under vacuum. mPEG-Mal was finally obtained.

CAAN-PTP-7 (1 mmol) and TCEP (2 mmol) were dissolved with DMF for 1 h in order to break the disulfide bond which could be oxidized by two sulfhydryl groups of cysteine. After that, 1 mmol mPEG-Mal was added into the solution and the mixture was stirred at room temperature for 4 h. The polymeric solution was subject to dialysis against 2 L deionized water at 4 °C for 48 h with 3500 Mw dialysis bag (Biotopped, Beijing, China). Then, the solution was lyophilized.

PTX (1 mmol), 2-propionic-3-methylmaleic anhydride (CDM, 1 mmol), EDC·HCl (3 mmol), and DMAP (0.1 mmol) were dissolved into dry DCM and stirred overnight under nitrogen atmosphere in room temperature. The reaction was monitored by thin-layer chromatography of DCM and ethyl acetate (3:1, v/v). Finally, the mixture was diluted with DCM, washed with HCl (0.01 M), saturated sodium bicarbonate and saturated brine in turn. After dried by sodium sulfate, filtered, concentrated and dried under vacuum, the crude product includes CDM-PTX that was obtained as a white solid.

PPP was constructed using PEG as the hydrophilic segment and PTP-7 as the hydrophobic segment. mPEG-PTP-7 (1 mmol), CDM-PTX (3 mmol), and DMAP (0.1 mmol) were dissolved in dry DMSO and stirred at 30 °C for 24 h under nitrogen atmosphere. The reaction was purified by dialysis against DMSO at room temperature for 48 h and further dialysis against PBS for 12 h with 3500 Mw dialysis bag. After washed with deionized water by ultrafiltration centrifuge tube (MWCO 10,000 Da), the solution was frozen at −80 °C for 4 h and lyophilized to afford the dried faint yellow powder of PPP. To obtain the PPP micelle, the solid powder of PPP was dissolved in THF and added dropwise to PBS and stirred overnight until THF volatilized completely. After filtrated by 450 nm filter, the PPP NPs were stored at 4 °C before use.^1^H NMR (400 MHz) (Bruker AVANCE DRX-400 NMR spectrometer, Bruker, Fällanden, Switzerland) was used to confirm the obtained compounds.

#### 2.2.2. Determination of critical micelle concentration (CMC)

Solution of Nile red in CH_2_Cl_2_ in brown bottles was evaporated under vacuum. PPP micelles with different concentrations ranging from 0.1 µg/mL to 150 µg/mL were added to the bottles and stirred in the dark for 12 h. A microplate instrument (Flexstation 3, Molecular Devices LLC, Sunnyvale, CA) was used to record the fluorescence of each solution at an emission wavelength of 620 nm and excitation wavelength of 579 nm. CMC of PPP micelle was calculated by plotting the ratios of intensity and concentration (Cai et al., 2020).

#### 2.2.3. Characterization of nanoparticles

The size, polydispersity index (PDI), and *ζ*-potential of PPP NPs were measured by dynamic laser scattering (DLS) (Zetasizer, Nano-ZS90, Malvern, Malvern, UK) and the morphology was observed by transmission electron microscopy (TEM) (Jeol Ltd., Akishima, Japan). In order to assess the stability, PPP NPs incubated in 10% FBS PBS solution were monitored by DLS for 13 days.

#### 2.2.4. *In vitro* hydrolysis of PPP

One milliliter aliquot of PPP NPs formulation containing 0.1 mg PTX in dialysis bags (MWCO = 3500 Da) was soaked in 29 mL PBS (pH 7.4 and 6.5) with 0.1% w/v Tween-80. The sample was incubated at 37 °C and shaking was done at 100 rpm. Two hundred microliters released mediums were collected and equal PBS was added at specific time points. The samples were added with 200 µL acetonitrile, then delivered with water/acetonitrile (v/v = 45/55) to HPLC system (Agilent, Santa Clara, CA) at a flow rate of 1.0 mL/min and detected at 227 nm by using a reversed-phase column (Inertsil C18, 5 µm, 4.6 mm × 250 mm, Shinjuku, Japan) at 27 °C (Cai et al., 2020).

#### 2.2.5. Cytotoxicity assay

MCF-7 cells, HCT116 cells, and 4T1 cells were seeded in 96-well plates at a density of 3 × 10^3^/well. After 12 h, cancer cells were incubated with free PTX and PPP micelles with a PTX concentration from 0.001 µg/mL to 10 µg/mL in 200 µL culture mediums for 72 h. One hundred microliters of culture medium containing 10 µL MTT (5 mg/mL) was added to each well for another 4 h in the dark. The absorbance was measured at 570 nm by a microplate reader (Flexstation 3, Molecular Devices LLC, Sunnyvale, CA). Comparing treated wells with controlled wells, the cell viability was analyzed as mean ± SD (*n* = 5). In order to confirm the enzyme responsiveness of AAN between the mPEG and PTP-7, MTT assay was used to indirectly observe the cytotoxicity of PTP-7 cleaved by legumain. One microgram of recombinant mouse legumain (Abcam, Cambridge, UK) was activated in a buffer solution (pH 4.5) which contains 50 mM critic acid, 1 mM DTT, and 1 mM EDTA (Zhou et al., 2017). Then, PP was dissolved in the legumain solution and incubated at 37 °C for 12 h. Next, culture medium containing 0.1–100 µg/mL of free PTP-7 or legumain treated mPEG-PTP-7 was added to cancer cells. After incubation for 24 h, the cell viability was calculated as above.

#### 2.2.6. Study of peptide–cell membrane interaction

To obtain PTP-7 completely cleaved by legumain, PP was dissolved in the buffer solution at a concentration of 5 µg/mL legumain and incubated at 37 °C for 12 h. After adjusting pH to 8, the solution was treated with 0.4 mmol of fluorescein isothiocyanate (FITC) (RHAWN, Shanghai, China) and stirred in the dark overnight to label PTP-7. MCF-7 cells were plated at 2 × 10^5^ cells per well in 12-well plates for 12 h and exposed to FITC-labeled PTP-7 at a concentration of 120 µg/mL PTP-7. The cells were washed with PBS for three times and the cell membranes were stained with Dil (10 µM) for 20 min. After being washed with PBS for three times to remove residual Dil, PTP-7 accumulation on cell membranes was investigated by fluorescent microscopy (Olympus IX73, Shibuya, Japan).

#### 2.2.7. Three-dimensional (3D) tomography of live cells

MCF-7 cells were seeded in a 35-mm glass-bottom dish for 24 h and then treated with PPP at a concentration of 20 µg/mL for 3 h. The live morphology of MCF-7 cells was detected using a 3D cell holographic tomography microscope (Nanolive 3D Cell Explorer, Tolochenaz, Switzerland) and analyzed by STEVE software. During imaging, cultivation environment was maintained with sufficient amount of CO_2_ at 37 °C.

#### 2.2.8. Cellular uptake

To verify cellular uptake of PPP NPs, 2 × 10^4^ cells of MCF-7 cells, HCT116 cells and 4T1 cells were seeded in 24-well plates. One milligram PPP and 0.01 mg coumarin (C6) were dissolved in THF, then the mixture was added dropwise to 1 mL PBS and stirred overnight. Filtered with a 220 nm filter to ensure the unencapsulated C6 was removed, C6-loaded PPP NPs (PPP-C6) were obtained and the C6 concentration was measured using a fluorescent microscopy (Olympus IX73, Shibuya, Japan) with excitation wavelength of 466 nm and emission wavelength of 504 nm. The encapsulation efficiency of C6 was 0.168% which showed the effective loading of PPP-C6. Next, the cells were treated with PPP-C6 at 10 µg/mL for 2 h. The medium was removed and the cells were washed with PBS for three times, fixed with 4% paraformaldehyde for 15 min. Hoechst 33342 was used to stain cell nuclei for 20 min. After washed with PBS for three times, cells were presented by fluorescent microscopy (Olympus IX73, Shibuya, Japan).

#### 2.2.9. Inhibition ability on tumor spheroid

MCF-7 cells and HCT116 cells were dispersed in culture medium containing 10% methylcellulose (0.24%, w/v) at a density of around 1 × 10^5^ cells/mL. Twenty microliters of the above medium was equably dropped on the lid of a 10 mm^2^ cell culture dish and incubated at 37 °C for 24 h. When the tumor spheroids were formed, they were removed to 96-well plates which were pre-coated with agarose. After incubation for 72 h, MCF-7 tumor spheroid was treated with PPP and free PTX at equivalent 0.1 µg/mL PTX and HCT116 tumor spheroid was treated with PPP and free PTX at equivalent 0.05 µg/mL PTX. The spheroid was observed for nine days and the diameter change of each spheroid was measured by Image J software (Bethesda, MD). Measured with maximum diameter (*d*_max_) and minimum diameter (*d*_min_), the tumor spheroid volume was calculated by the following equation: *V*=(π×*d*_max_×*d*_min_)/6, and the negative control tumor spheroids grew up in culture medium.

#### 2.2.10. Pharmacokinetic evaluation

The pharmacokinetics of PPP NPs was tested on female ICR mice (∼5 weeks) compared with the clinically used Taxol. The mice were randomly divided into two groups (*n* = 4) and intravenously injected with PPP NPs and Taxol at a PTX dosage of 7.5 mg/kg. At 0.25, 0.5, 1, 2, 4, and 6 h, blood of postorbital venous plexus was collected by capillaries into 1.5 mL heparinized EP tubes and centrifuged immediately for 10 min at 4 °C, 5000 rpm. Then, 50 μL of serum was mixed with 150 μL acetonitrile and the mixture was centrifuged for 10 min to precipitate the proteins at 4 °C, 5000 rpm. The supernatant was taken out and pretreated with 1 M hydrochloric acid overnight to completely release the PTX of PPP. After filtered with a 220 nm filter, the samples were analyzed by HPLC using water/acetonitrile (v/v = 45/55) as the mobile phase. The analyzed flow rate was 1 mL/min and detected at 227 nm.

#### 2.2.11. *In vivo* tissue biodistribution studies

The *in vivo* tissue biodistribution studies of PPP NPs were carried out in female BALB/c nude mice. To establish MCF-7 tumor-bearing model in the right flank of mice, each mouse was injected with 1 × 10^7^ cells. The tumor volumes were calculated by the following equation: *V*=length × width^2^/2. When the tumor grew up to ∼200 mm^3^, PPP NPs and Taxol (10 mg/kg PTX equiv.) were injected intravenously. After injection for 8 h, the heart, liver, spleen, lung, kidney, and tumor of each mouse were dissected, weighed, and homogenized with three times volumes of deionized water. The homogenization and acetonitrile were mixed at a ratio of 1:3. After vortexed for 10 min, the mixture was centrifuged at 5000 rpm, 4 °C for 10 min. The supernatant was collected and dried by vacuum pump. Next, the residuum was redissolved with 80 μL of mobile phase (water/acetonitrile: 45/55) and treated with 1 M hydrochloric acid to completely release the PTX. The HPLC analyzed method was consistent with the method described above.

#### 2.2.12. Antitumor efficacy and safety evaluation

Female BALB/c nude mice were (∼5 weeks) implanted with 1 × 10^6^ 4T1 cells in the right flank. The 4T1 tumor-bearing mice were casually divided into three groups (*n* = 4) when tumor volume reached about 150 mm^3^. All mice were intravenously injected with 200 µL PBS, Taxol, and PPP NPs (PTX, 7.5 mg/kg) every two days for five times. Tumors diameter and body weights were recorded every two days and the relative tumor growth was expressed as a rate of volume and initial volume. When all treatments were accomplished, three groups of mice were euthanized for dissecting. Tumors and major organs were collected, washed, and fixed with paraformaldehyde for further histological examinations.

#### 2.2.13. Statistical analysis

All data analyzed for statistic were performed with GraphPad Prism (San Diego, CA) as mean ± SD. One-way ANOVA was used to evaluate the significant comparison between two groups. Statistical significance was indicated as *p<.05 and high significance was marked as **p<.01.

## 3. Results and discussion

### 3.1. Characterization of PPP NPs

**Figure 2.**
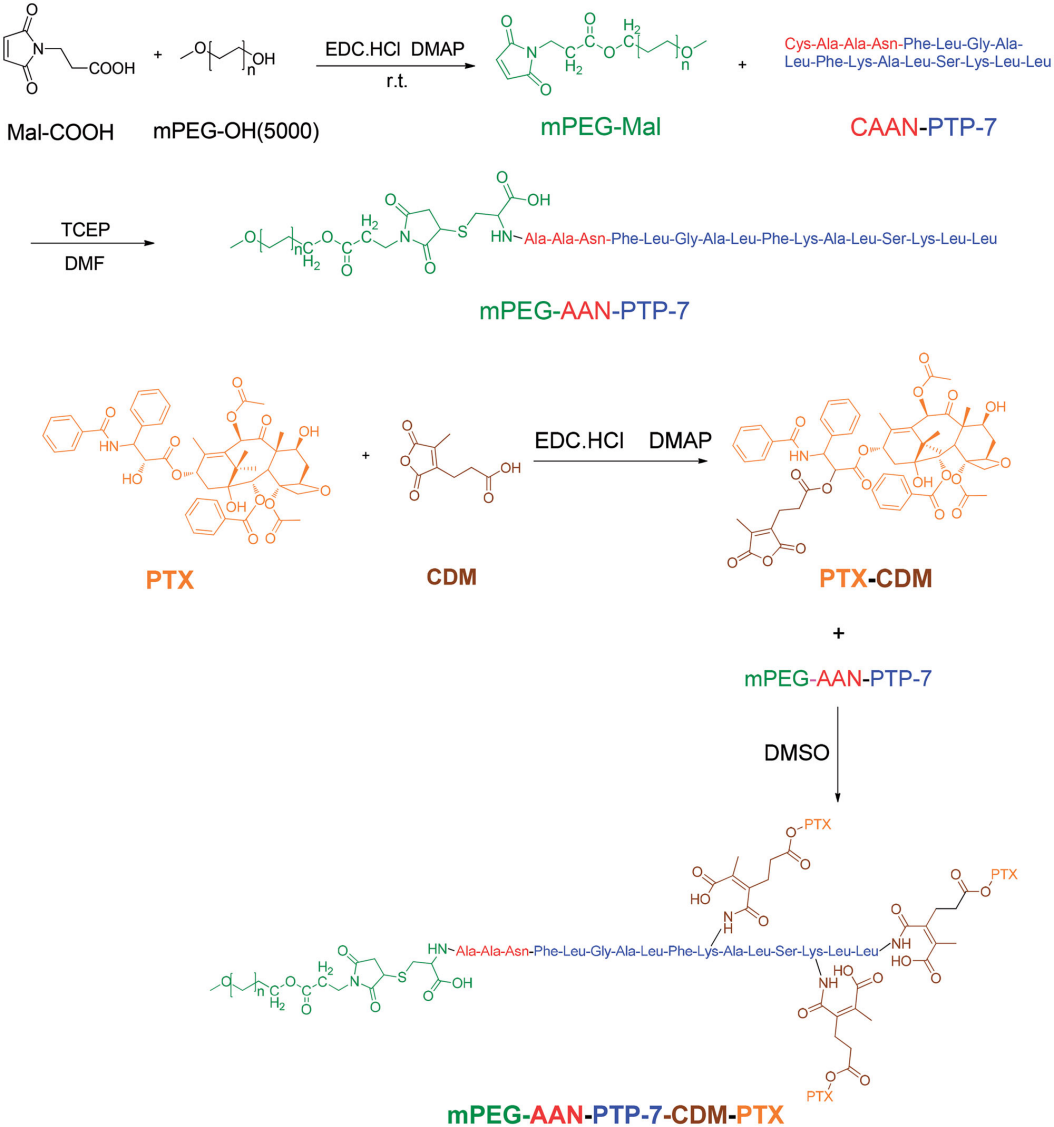
Synthetic route of mPEG-AAN-PTP-7-CDM-PTX (PPP).

Synthesis route of PPP which consisted of mPEG-OH, Mal-COOH, CAAN-PTP-7, CDM, and PTX is shown in Figure 2. To maximize the interactions between PTP-7 and tumor cells, pro-peptide CAAN-PTP-7 (CAANFLGALFKALLAAN) was prepared by modifying with cysteine–alanine–alanine–asparagine (CAAN), a particular legumain substrate, to the phenylalanine of PTP-7. First, mPEG5000-Mal was synthesized by esterification using mPEG5000-OH and the yield of mPEG5000-Mal was 63%. Fig. S1-A indicates the successful connection of maleimide group (*δ* = 6.7 ppm) to mPEG. Then mPEG-Mal was connected with CAAN-PTP-7 by addition reaction between the double bond of maleimide group and the sulfhydryl of cysteine (Fig. S1-B) and the yield of mPEG-PTP-7 was 78%. mPEG5000-OH was served as hydrophilic segments to prolong the circulation in blood and PTP-7 was served as hydrophilic segments. For PPP synthesis, PTX was reacted with CDM to prepare PTX-CDM and the yield of product was 51%. As shown in Fig. S1-D, new peaks appeared at 2.1 ppm and 2.8 ppm in comparison with PTX spectrum in Fig. S1-C. Then, the product was further coupled to mPEG-PTP-7 through a ring-opening and addition reaction between the CDM anhydride residue and the amino groups of PTP-7. The yield of PPP was 62% and the resultant amide bond would be hydrolyzed in the acid tumor microenvironment and then liberate PTX. The 1H NMR of PPP (Fig. S1-E) showed new hydrogen spectrum peaks of CDM-PTX compared to 1H NMR of PP (Fig. S1B) which indicated a 14% PTX loading content of PPP. Calculated by 1H NMR of PPP, the molecular weight of PPP was 7074.38.

**Figure 3.**
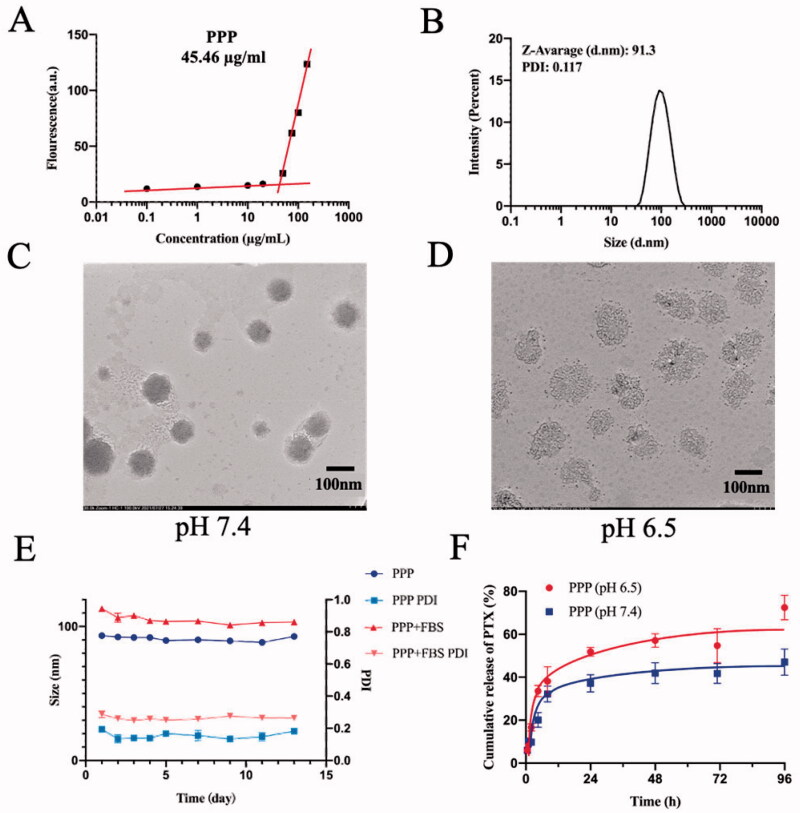
Characterization of PPP NPs. (A) The CMC value of PPP. (B) Diameter and PDI distribution of PPP NPs. (C) TEM images of PPP at pH 7.4. Bar = 100 nm. (D) TEM images of PPP at pH 6.5. Bar = 100 nm. (E) The stability of PPP in 10% FBS and PBS respectively. (F) *In vitro* PTX release curves at pH 7.4 and pH 6.5, respectively.

The CMC is a critical property for micelles as it indicates the polymer’s capacity to form micelles in aqueous solutions. As shown in Figure 3(A), the CMC of PPP was 45.46 µg/mL which stated that PPP was easy to transform micelles.

The results of DLS displayed that the average particle size of PPP was about 91.3 nm with a narrow size distribution, which was the proper size to penetrate effectively into the tumor site due to EPR effect (Figure 3(B)) (Zhang et al., 2018). The *ζ*-potential of PPP was approximately −3.97 mV (Fig. S2) which imparted longer blood circulation time to PPP. To test the stability of NPs in the mimicked physiology, the PPP NPs were incubated in PBS with 10% FBS and its size and PDI were observed by DLS. The average size and PDI were hardly changed in 13 days which indicated an excellent stability of PPP (Figure 3(E)).

As indicated in Figure 3(C,D), PPP NPs were spherical and uniform in normal condition and the average particle size of PPP in the TEM image measured by Image J software (Bethesda, MD) was about 90.8 nm, while the nanostructure became less compact and transformed into a lysis that consisted of mPEG-PTP-7 in acidic condition. The results preliminaries verified the pH-responsive of PPP.

### 3.2. *In vitro* release of PPP NPs and dual-responsive assays

*In vitro* release of PTX underwent in two different pH dissolve mediums (pH 6.5 and 7.4) to further reveal the acid-responsiveness of resultant amide bond. As shown in Figure 3(F), PPP NPs in a weak acid medium presented a relatively quicker release with a 72.5% cumulation at 96 h than PPP in normal medium (47.4%). On a consequence of the different pH between normal tissues and tumor microenvironment, NPs with less cumulation release at pH 7.4 were favorable to the stability in blood circulation and low toxicity to normal tissues, while greater release of PTX at pH 6.5 pointed out an accurate targeting in tumor.

**Figure 4.**
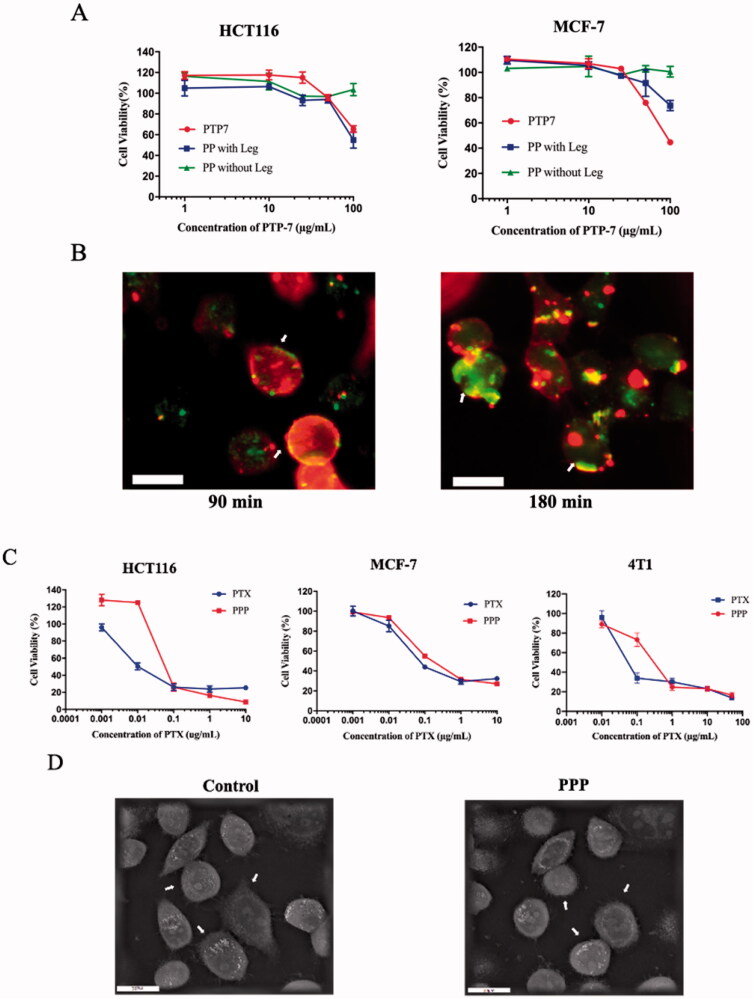
(A) Legumain responsiveness of PP: cell viability of HCT116 and MCF-7 after incubating with free PTP-7, PP in presence or in absence of legumain. (B) Kinetics of PTP-7 interaction with MCF-7 cells: cells were incubated with FITC-labeled PTP-7 for 90 min and 180 min. Magnification: ×20. Bar = 25 µm. (C) Cell viability of HCT116, MCF-7, and 4T1 after incubating with free PTX and PPP for 72 h. (D) Holotomographic 3D images of cell morphology in MCF-7 cells treated with 20 µg/mL PPP for 3 h. Bar = 20 µm.

The cytotoxicity of PTP-7 cleaved by legumain was measured by MTT assay to determine enzyme responsiveness of mPEG-PTP-7 (PP) (Figure 4(A)). Compared with free PTP-7, PP incubated with legumain showed considerable cell-killing ability on MCF-7 and HCT116 cells. In contrast, PP in the absence of legumain had little influence on cell viability. The inhibitory effects revealed that PTP-7 could be released from PP and produced membrane lytic activities in the tumor environment.

### 3.3. Peptide–cell membrane interaction

To further assess the cytotoxicity mechanism of PTP-7, interactions between FITC-labeled PTP-7 and Dil-labeled cell membranes were studied (Figure 4(B)). After 90 min of incubation, green fluorescence (FITC-labeled PTP-7) appeared on the cell surfaces and some cell membranes color changed from red to yellow (a mixture of green fluorescence and red fluorescence), which indicated that PTP-7 formed an extensive layer on cell surfaces and inserted into the lipid bilayer by a detergent-like manner. As the incubation time increased to 180 min, cell membranes were severely damaged and peptide aggregates could be clearly seen in the cytoplasm, demonstrating the cell lysis mechanism of PTP-7.

### 3.4. Cell cytotoxicity and cellular uptake

The cytotoxicity of PPP in MCF-7, HCT116, and 4T1 cells was investigated by MTT assay (Figure 4(C)). The half maximal inhibitory concentration (IC_50_) of PPP NPs and free PTX was calculated to be 0.066 and 0.028 µg/mL on MCF-7 cells, 0.044 and 0.006 µg/mL on HCT116, 0.212 and 0.01 µg/mL on 4T1 cells which indicated a lower cell growth inhibition ability than free PTX. The result may be owing to the extremely stable property of PPP and low legumain expression *in vitro* leading to incomplete release of PTX and PTP-7 (Edgington et al., 2013). Moreover, the holographic microscopy studies showed the changes in cellular morphology of MCF-7 cells after treated with PPP for 3 h. Notably, PPP led to the morphological features of apoptosis such as shrinkage, losing contact with neighboring cells and floating relative to control (Figure 4(D)).

**Figure 5.**
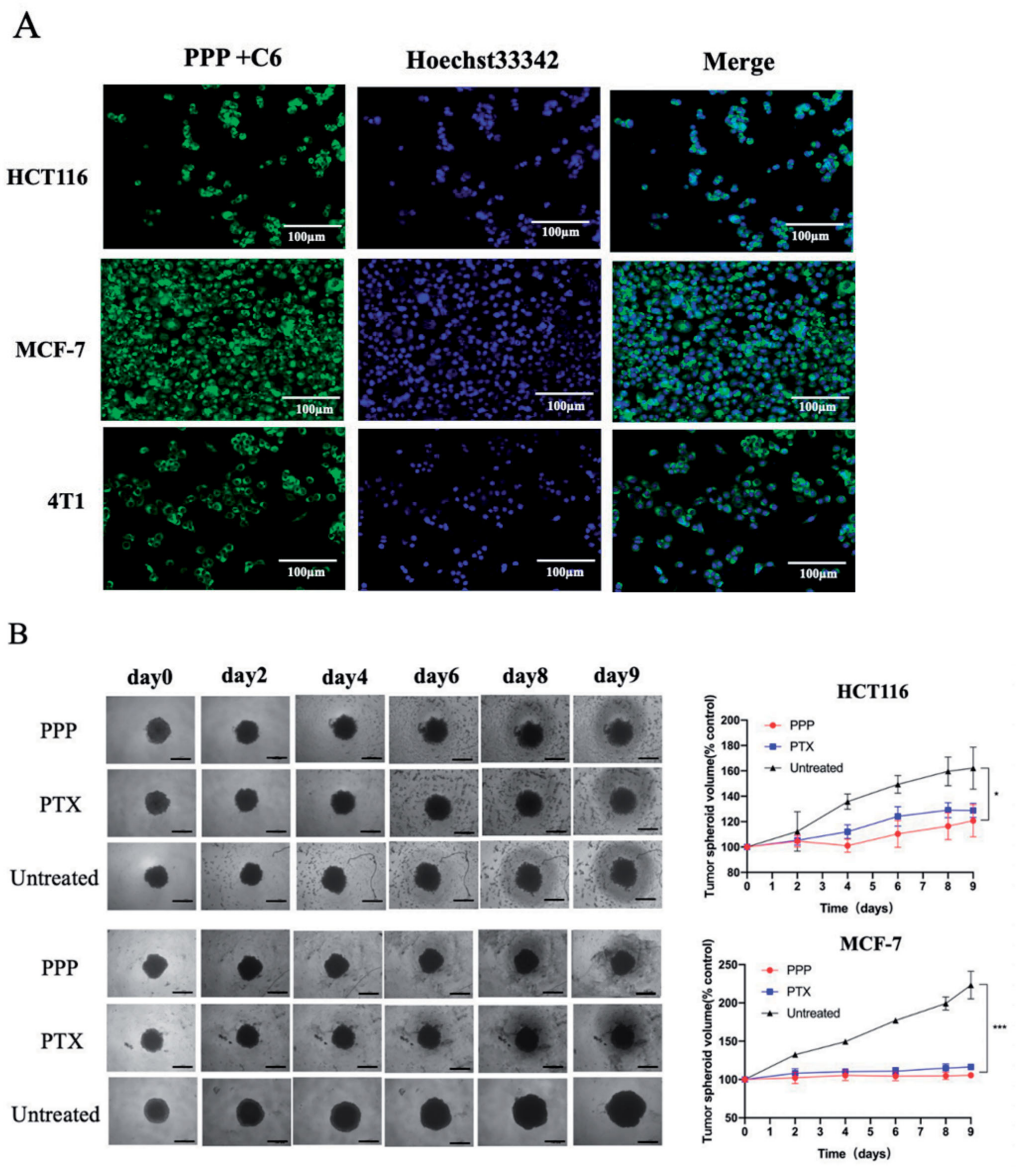
(A) Cell uptake images of PPP in HCT116, MCF-7 cells, and 4T1 cells after incubating with PPP-C6 at 10 µg/mL for 2 h. Nuclei and PPP-C6 were labeled as blue fluorescent and green fluorescent, respectively. (B) Representative tumor spheroids images and tumor spheroid volumes growth rate of HCT116 and MCF-7. Magnification: ×4. Bar = 500 µm.

Next, cell uptake of PPP NPs was studied against MCF-7, HCT116, and 4T1 cells by fluorescent microscopy. As shown in Figure 5(A), judging by the blue fluorescence, the cytoplasm was stained with a distinct green fluorescence, which means PPP NPs could be endocytosed by cells after incubated with PPP-C6 NPs for 2 h. The results clearly showed the rapid cell association with PPP NPs.

### 3.5. Inhibition of tumor spheroid growth

The tumor spheroids were used to mimic tumor microenvironment (Sung & Beebe, 2014) and the spheroids volume change was measured to further evaluate the cell viability (Figure 5(B)). The PPP treatment led to a 25.49% inhibition of HCT116 spheroids while free PTX treatment was 20.51% suppression at 9th day. Meanwhile, the inhibition of PPP NPs and PTX was 52.68% and 47.88% on MCF-7 spheroids, respectively. At equivalent PTX concentration, the tumor spheres treated with PPP demonstrated slightly effective inhibition compared to the spheres treated with free PTX. In a way, we could own part of inhibition of PPP to PTP-7 cleaved by legumain.

### 3.6. Pharmacokinetics and biodistribution

**Figure 6.**
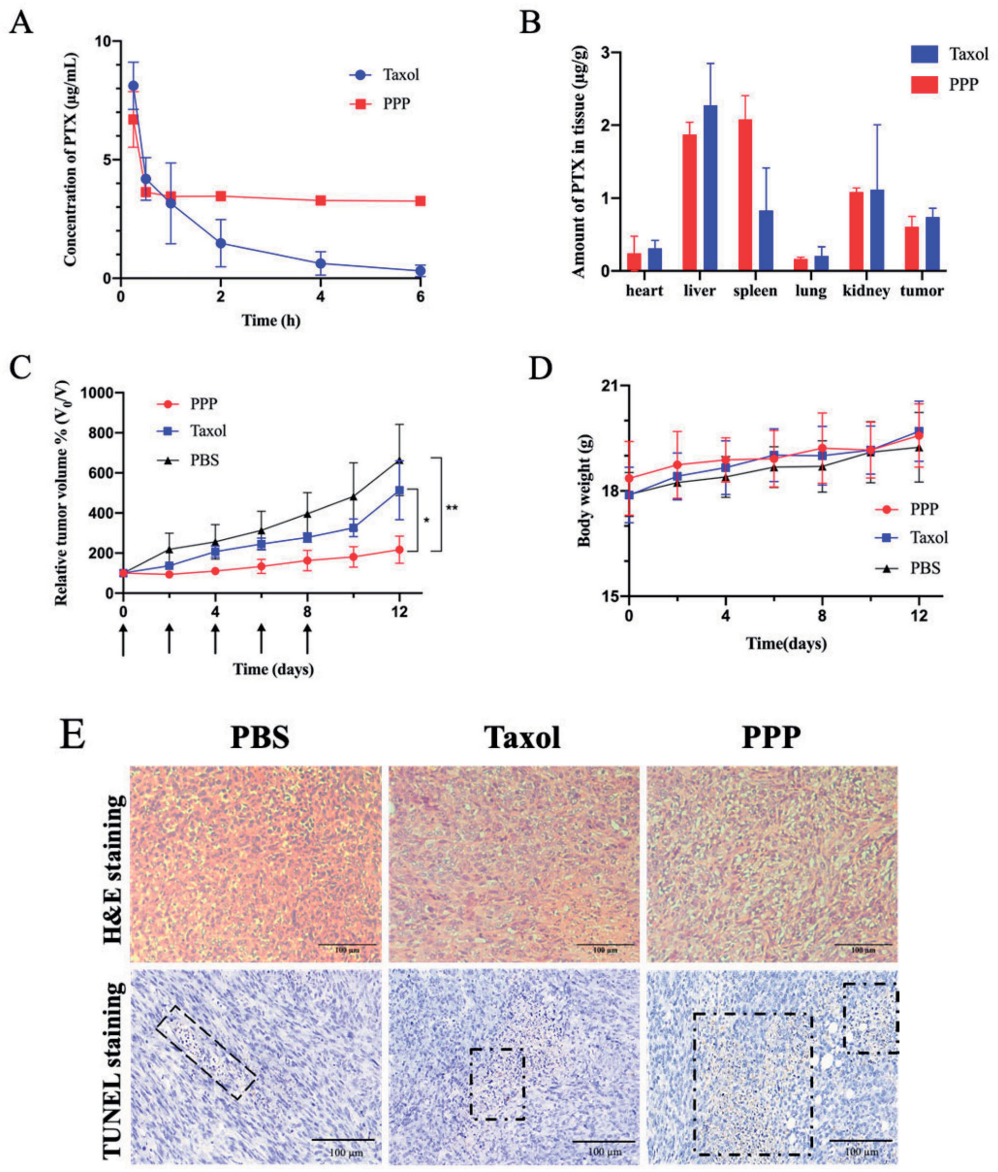
(A) Plasma concentration of PTX in ICR mice after administration of Taxol and PPP (7.5 mg/kg PTX equiv., *n* = 5). (B) The biodistribution of PTX in BALB/c nude mice bearing MCF-7 tumor at 8 h after administration of Taxol and PPP (10 mg/kg PTX equiv., *n* = 3). (C–E) Antitumor efficacy of Taxol and PPP. Relative tumor volume change with treatment for 12 days in (C) and the arrows signify the time of intravenous administration, body weight changes of BALB/c nude mice bearing 4T1 tumor during treatment in (D) and the H&E and TUNEL assay of tumor tissues from three groups in (E). Data are described as mean ± SD (*n* = 4, *p<.05, **p<.01). Bar = 100 µm.

Recent researches have established that PTX encapsulated in formulation still have the disadvantage of rapid blood elimination (Koudelka & Turánek, 2012; Nehate et al., 2014). Pharmacokinetics of PPP NPs and Taxol were tested on female ICR mice (6–8 weeks) to determine whether NPs can prolong the blood circulation of PTX. 7.5 mg/kg PTX dosage of Taxol and PPP were administrated through tail vein injection. As Figure 6(A) and Table 1 show, compared with Taxol, PPP NPs displayed a dramatically longer blood circulation time. As displayed in Table 1, the circulation half-life and concentration–time curve (AUC_0–∞_) of the PPP were calculated to be 51.7 h and 134.3 mg L^−1^ h^−1^, which were 56.5 and 7.5 times as much as that of Taxol. We speculated that superior body accumulation of PPP was attributed to its enhanced solubility, stability, and stealth performance of pegylated nanoparticles surface (Indoria et al., 2020; Kim et al., 2020).

**Table 1.** Pharmacokinetic parameters of PTX and PPP injected in ICR mice bearing MCF-7 tumor (*n* = 3).

Parameters (unit)	PPP (i.v. 7.5 mg/kg)	Taxol (i.v. 7.5 mg/kg)
*t*_1/2α_ (h)	0.056	0.03
*t*_1/2β_ (h)	51.659	0.914
CL (L h^–1^ kg^–1^)	0.056	0.416
AUC_(0–∞)_ (mg L^–1^ h^–1^)	134.287	18.011
*C*_max_	6.702	7.424

*t*_1/2α_: distribution half-life; *t*_1/2β_: elimination half-life; AUC_(0–∞)_: area under curve; CL: plasma clearance; *C*_max_: peak concentration.

The biodistribution of PPP in heart, liver, spleen, lung, kidney, and tumor was further investigated in MCF-7 tumor-bearing BALB/c nude mice (Figure 6(B)). The PPP treatment showed dramatically high accumulation in spleen than Taxol treatment probably owing to its long blood retention and subsequently nonspecific uptake by macrophages in spleen through the mononuclear phagocyte system (Zhang et al., 2016). Moreover, PPP NPs and Taxol showed high accumulation in liver on account of strong clearance of liver phagocytic cells (Tsoi et al., 2016). Due to the lack of targeted ligand modification, PPP NPs exhibited no significant difference accumulation in tumor to Taxol.

### 3.7. *In vivo* antitumor efficacy and safety evaluation

In view of the performance of PPP NPs *in vitro* and superior pharmacokinetic *in vivo*, the *in vivo* anti-cancer efficacy was performed on 4T1 tumor overexpressing legumain proteases *in vivo* (Liu et al., 2014; He et al., 2018). It is observed that all of tumor volumes were suppressed with treatment of PPP and Taxol compared to PBS (Figure 6(C)). Noted that PPP NPs exhibited more distinct inhibition than Taxol, and average relative tumor volumes of PPP NPs and Taxol treated mice reached to 217% and 514% on day 9 compared to the first day, respectively. The satisfactory antitumor effects of PPP NPs should be put down to the drugs programmed release and the co-therapy of PTX and PTP-7. When PPP NPs were carried to tumor tissue, the acid-responsive CDM linker was triggered in the weak acid tumor microenvironment to release PTX. Next, PTP-7 peptide contained in PPP NPs was specifically activated by highly expressed legumain, thereby achieving the lytic efficiency on tumor cells.

*In vivo* therapeutic safety of PPP NPs was observed by monitoring the changes of mice body weight. Compared with PBS group, none of mice treated with PPP NPs or Taxol showed significant body weight loss (Figure 6(D)) indicating no systemic toxicity for both groups. Furthermore, TUNEL of tumors and H&E staining of major organs at the end of treatments were further used to evaluate therapeutic safety (Figure 6(E)). H&E staining indicated that cell morphology and nuclear in tumors were almost intact with PBS treating group. On the contrary, PPP NPs treated group displayed the largest quantity of cell apoptosis compared to Taxol and PBS groups which can be seen more noticeable in TUNEL images. It is worth mentioning that some PBS treated mice suffered from splenomegaly, secreted more inflammatory cells and Taxol treated group showed mild shriveled glomerular and myocardial fiber breakage, while the PPP treated mice was normal. Beyond that none of mice presented significant tissue damages and abnormal behaviors manifesting non-toxicity of PPP (Figure S3).

## 4. Conclusions

In summary, we successfully constructed a PTP-7 conjugated PTX nanoparticles to accomplish the cascade drug release in unique tumor microenvironment. The obtained PPP NPs possessed perfect stability *in vitro* and had longer blood circulation *in vivo* that were desirable as NDDS. When delivered to tumor microenvironment, PPP NPs could be activated in tumor extracellular matrix or intracellular lysosomes. The specificity of acid pH-stimuli and legumain-sensitive endows PPP NPs with precise drug release in the tumor site and synergistic therapy of PTX and PTP-7. Afterwards, the enhanced therapeutic efficacy on tumors produced by synergistic treatment of PTP-7 and PTX has justified the applicability of dual-responsive strategy. Given above, the rational design of PPP NPs has transmitted a strategy for environment-responsive drug delivery of overcoming drug barriers and also provided ideas of therapeutic peptide for broader clinical application.

## Supplementary Material

Supplemental Material
