# Supplementary material for: Legumain/pH dual-responsive lytic peptide–paclitaxel conjugate for synergistic cancer therapy
Source: Drug Deliv. 2022 May 31;29(1):1764–75. doi: 10.1080/10717544.2022.2081380 (PMC9176665; doi:10.1080/10717544.2022.2081380)
Supplement: Supplemental Material [file IDRD_A_2081380_SM9676.docx]

**Legumain/pH Dual-Responsive Lytic Peptide-Paclitaxel Conjugate for** **Synergistic Cancer Therapy**

Shanshan Zheng, Yue Cai, Yulu Hong, Yubei Gong, Licheng Gao, Le Li, Xuanrong Sun^[[1]](#footnote-1)^*

Collaborative Innovation Center of Yangtze River Delta Region Green Pharmaceuticals Zhejiang University of Technology, Hangzhou 310006, China

***Cells and animals***

The cell lines were purchased from China Center for Type Culture Collection (Wuhan, China). MCF-7 and HCT116 cells were cultivated in DMEM and 1640 with 10% FBS and 1% PS. 4T1 cells were cultivated in 1640 containing 1% glutaMAX^TM^-1, 1% MEM NEAA, 1% sodium pyruvate, 10% FBS and 1% PS. All cell lines were incubated in incubator at 37℃ and 5% CO_2_. All qualified mice were purchased from Hangzhou Medical College and fostered in laboratory animals and all animal experiments followed the ethical requirements set by the ethics committee of Zhejiang University of Technology (20210511034).


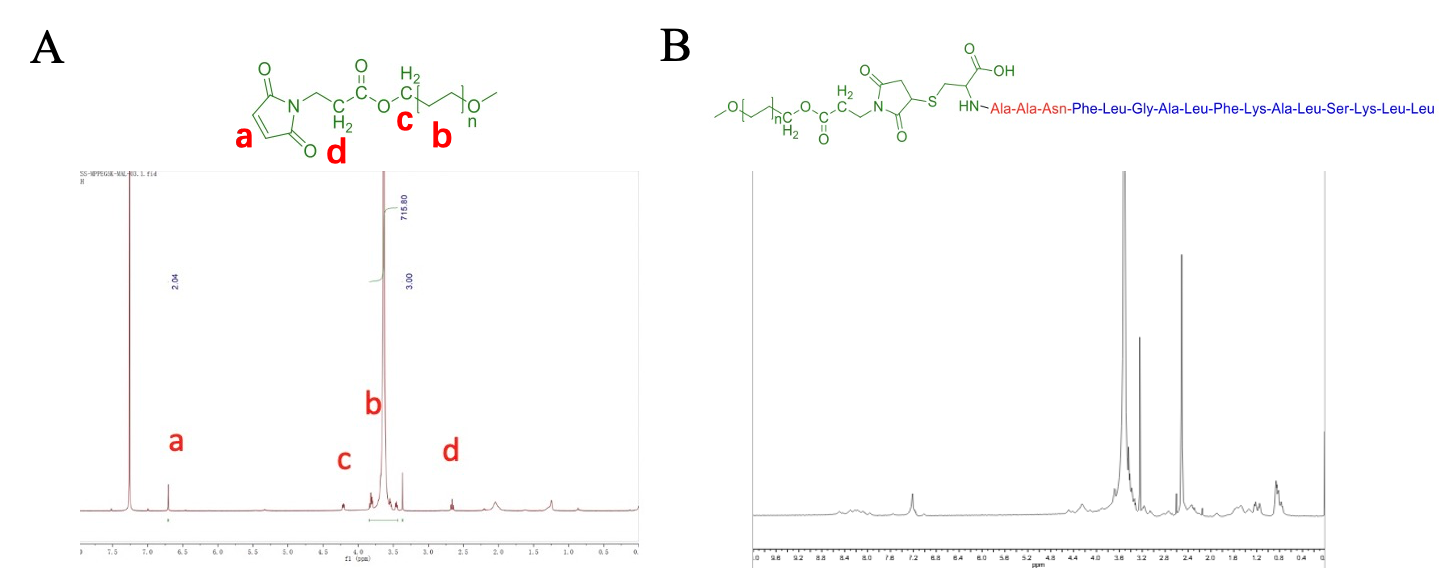


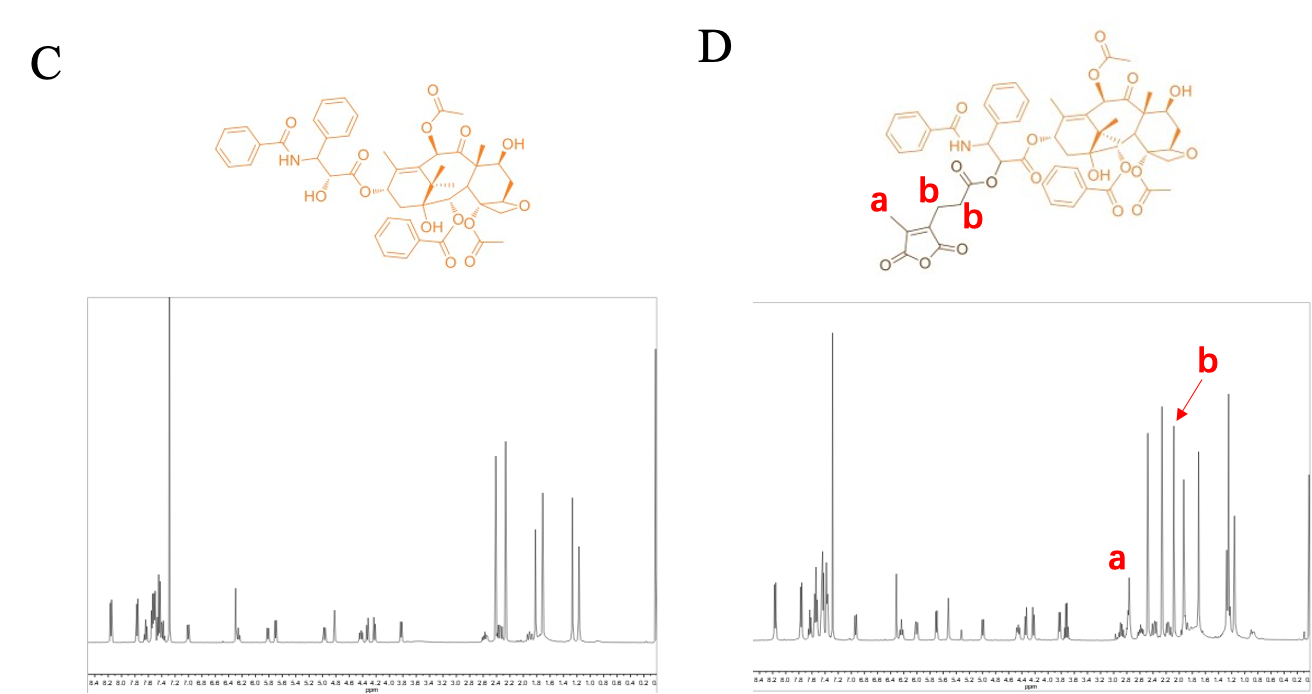


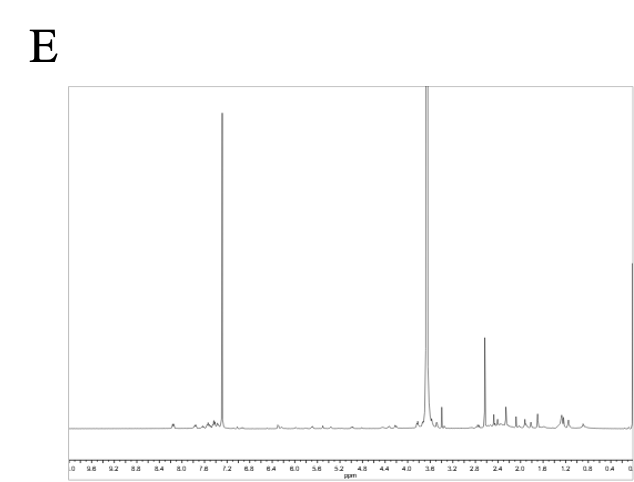


**Figure S1.** (A) ^1^H NMR spectrum of mPEG-MAL in chloroform. (B) ^1^H NMR spectrum of mPEG-PTP-7 in DMSO. (C) ^1^H NMR spectrum of PTX in chloroform. (D) ^1^H NMR spectrum of CDM-PTX in chloroform. (E) ^1^H NMR spectrum of PPP in chloroform.


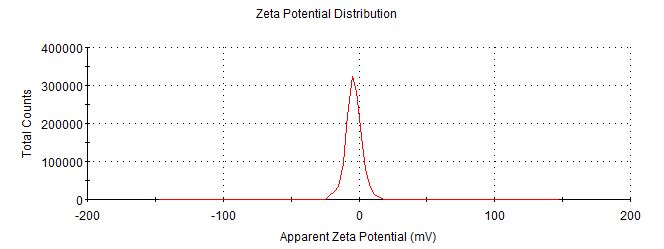


**Figure S2.** ζ-potential of PPP.


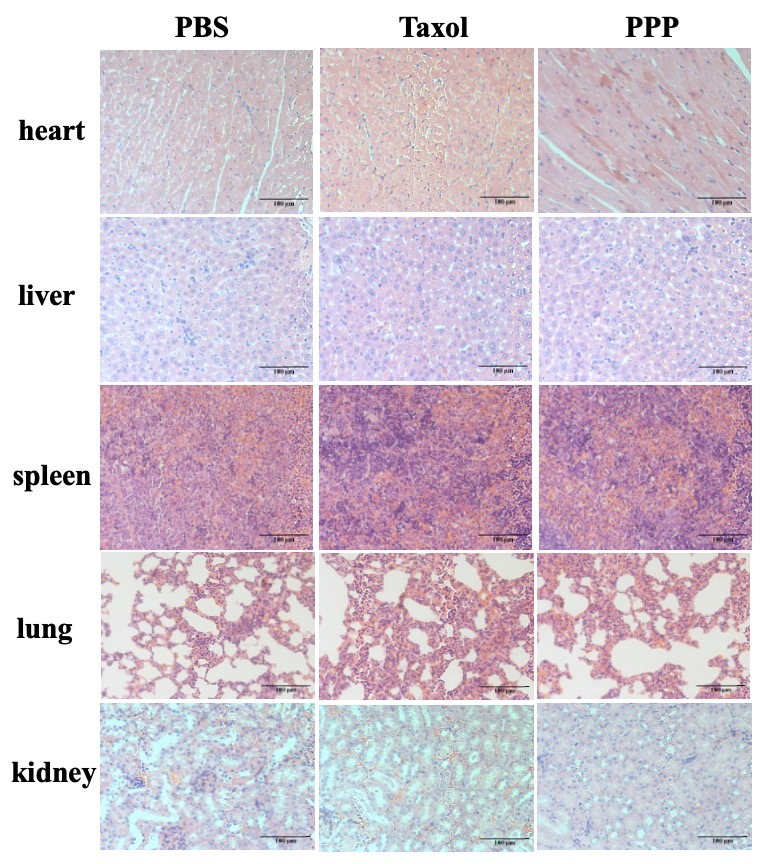


**Figure S3.** Biosafety evaluation of major organs with treatment of Taxol, PPP NPs and PBS by H&E staining. Bar = 100 µm.

1. * Corresponding author. Email: sunxr@zjut.edu.cn [↑](#footnote-ref-1)
